# Radiological Study on the Evolution of the Biomaterial’s Image (Shape, Density, Vertical Dimensions) after the Lateral Sinus Lift

**DOI:** 10.3390/dj11120273

**Published:** 2023-11-29

**Authors:** Dragos Epistatu, Octavian-Marius Dinca, Cristian Vladan, Cristina Padurariu, Iulian Damian, Sorin Hostiuc

**Affiliations:** 1 Department of Dental Radiology, Dental Medicine Faculty, “Carol Davila” University of Medicine and Pharmacy, 37 Dionisie Lupu Str., 020021 Bucharest, Romania; 2 Department Oral and Maxillofacial Surgery, “Dan Theodorescu Hospital”, “Carol Davila” University of Medicine and Pharmacy, 37 Dionisie Lupu Str., 020021 Bucharest, Romania; octavian.dinca@umfcd.ro (O.-M.D.); cristi.vladan@umfcd.ro (C.V.); lavinia.padurariu@umfcd.ro (C.P.); 3 Department of Oral Rehabilitation, Ovidius University, 124 Bd. Mamaia, Aleea Universitatii nr.1, 900527 Constanta, Romania; iulian.damian@365.univ-ovidius.ro; 4 Department of Legal Medicine and Bioethics, “Carol Davila” University of Medicine and Pharmacy, 37 Dionisie Lupu Str., 020021 Bucharest, Romania; sorin.hostiuc@umfcd.ro

**Keywords:** lateral sinus lift, different biomaterials, radiological density, shape of the upper plane, vertical dimensions, contraction of biomaterial

## Abstract

The aim of this study is to evaluate the long-term changes of sinus lift material. Materials and methods: We included a total number of 35 patients (20 men and 15 women), between 32 and 80 years old, evaluated on a timeframe of up to 11.6 years. Diverse biomaterials were used (allograft, xenograft, alloplastic, combinations of them), with autologous bone in some cases. Results: The appearance of the top plane of the bone over time took a large dome shape (36% of cases), a linear shape (32% of cases), an irregular shape (23% of cases), or had micro domes above the implants (7%). No significant differences were found between the groups regarding age. The radiological density of the biomaterial tended to equalize that of the native bone. The final vertical dimensions seemed to be independent of the initial native bone height but seemed to be correlated with the amount of applied biomaterial. For the study group, the biomaterial contracted on average by 10% for the maximum height (H max) and 20% for the minimum height (H min), which can explain the tendency of the upper border of the biomaterial to curve. The annual H max contraction ranged from −0.09 to +0.18 with a mean value of 2.67% (SD = 0.04, CI: [0.011, 0.041]). The median value was 1.8%. The annual H min contraction ranged from −0.24 to +0.24, with a mean value of 4.33% (SD = 0.07, CI: [0.021, 0.065]). The median value was 3.59%. There were no statistically significant gender differences (Mann–Whitney U, *p* = 0.483, *p* = 0.642). The additional application of biomaterial together with the implants seemed to have a beneficial effect on the final vertical dimension of the bio-transformed material.

## 1. Introduction

After the extraction of the maxillary teeth from the lateral area, the phenomenon of vertical resorption of the alveolar process, together with the pneumatization of the sinus, leads to a decrease in the height of the edentulous ridge up a size of minimum 1–3 mm, an instance in which there still is a therapeutic solution [1]. The application of implants below the limit of 5–6 mm of the bone height is generally performed by raising the Schneider’s membrane using a lateral bone window (known as the “sinus lift procedure”—Tatum 1976) and by applying a biomaterial inside the sinus [1]. However, there are other options that have been reported. For example, some researchers did not use biomaterial but only a repositioning of the membrane, the resulting space being filled by the blood clot [2]. Also, the “osteotome-based technique” (Summers 1994), also called the “crestal sinus lift” procedure, can generally be used for an up to 4 mm elevation of the sinus membrane. There are also reported variants with larger sizes [3] and multiple modified variants compared to the initial description [4,5,6]. 

It is known that all biomaterials used for a sinus lift (SL) graft suffer a gradual biotransformation. Examining the morphology, density, and absorption of the biomaterials in the sinus graft is essential for the understanding of how they change over time until becoming bone structure, providing a living tissue as a support for implants. A meta-analysis published in 2019 that evaluated 1443 histomorphometrically analyzed biopsies, showed that for the first six months after intervention, autologous bone alone as grafting material gave the best results regarding the real formation of new bone (usable ridge height). It also showed that other types of biomaterials may be used, especially in combination with autologous bone, for “superior histomorphometric” results [7]. There are also numerous other studies analysing the success of using other biomaterials, reporting different percentages of biotransformation [8,9,10,11,12,13].

Even after bone has been formed, living tissue remodels itself throughout life. Dimensional differences observed over time are called contraction (shrinkage) in the literature, even if it is not a simple contraction but a more complex process. We could normally consider that the graft will persist almost in the same shape as it is at 6 months after the grafting procedure [14]. On the contrary, we can believe that it will be continuously resorbed over 5–10 years [15]. The literature contains partial and sometimes contradictory information on the subject we are addressing. One recent study (2022) suggests that vertical graft changes are fairly independent of the baseline graft height [14]. Another study (2020) concluded that the volume of the graft and its characteristics can influence the remaining bone volume over time, considering the shrinkage of the biomaterial [16].

We need to analyze many cases treated with various materials, especially after long periods of time after completing the treatments. 

The radiological density of biomaterials and its evolution has not been well objectified in the literature. It is very useful to have a research-based guideline for radiological density analysis that is much more accessible to practitioners than histomorphometry. Also, we must define what are the normal variants of the graft’s shape. Even though 3D CBCT exploration is more accurate, 2D radiographs remain a useful and comprehensive tool for recent retrospective studies comparing the evolution of the sinus graft image [14,16].

In clinical practice, only the test of time and the success of prosthetic implant treatments remain to confirm the success of the sinus bone graft in each individual case. Any clinician would like to know more precisely what the lifespan of implants is after a sinus lift, but objective criteria must be found.

That is why the purpose of our study is to answer the following questions: (1) what is the normal appearance of sinus grafting material after several years and (2) what dimensional changes are normal during this period?

We started our study based on three main null hypotheses:

1. The upper limit of the sinus graft takes a flat shape over time.
2. The density of the graft eventually becomes equal to the native bone.
3. The SL graft resorbs a lot after several years, approaching the original dimensions of the native bone.

## 2. Materials and Methods

### 2.1. Study Design

This study was retrospective and observational and was performed using radiographs of already-treated patients, with the consent of the Institutional Review Board. The patients’ treatments were performed in the same private dental clinic in Bucharest, Romania, by different surgeons who followed the same standard protocol and had the same supervisor. The informed consent for the treatments and the retrospective study data evaluation and publishing have been obtained from all included subjects. All patients had posterior edentulous areas that required sinus lift procedures because the alveolar bone was not of sufficient height to stabilize all the implants.

The patients were included in the study if they met the necessary health conditions to be able to undergo the surgical procedures. All patients were clinically examined and underwent initial blood tests, orthopantomography (OPG), and cone beam computed tomography (CBCT) on the area of the maxillary sinuses. They also completed the declarations regarding possible ailments and current medication. The exclusion criteria were represented by significant systemic diseases (hepatic, renal, serious cardiac failure, certain allergies, metabolic diseases, autoimmune diseases, and certain endocrine diseases that could influence bone metabolism). Smoking patients were not excluded. However, the patients who, at the initial CT scan, showed signs of sinus pathology, including the thickening of Schneider’s membrane over 2 mm, sinus polyps, cysts, mucoceles, etc., were treated surgically before. On the occasion of the sinus lift (SL) intervention, the condition of the healthy membrane was checked intraoperatively.

### 2.2. Surgical Procedure

The interventions were made through the sinus lateral wall, respecting the minimum invasiveness criteria. The sinus membrane was checked intraoperatively for suppleness and strength. Possible small perforations of the membrane up to 5/5 mm were allowed, in which cases, a special thin collagen membrane was used. The applied biomaterials were diverse, starting with allograft, xenograft, alloplastic material, and combinations. Whenever possible, a quantity of autologous bone was used, usually harvested from the maxillary tuberosity. A thicker collagen membrane was applied over the sinus window, from various manufacturing companies, and careful suturing of the flap was performed. 

All patients received postoperative antibiotic and anti-inflammatory treatment for seven days, usually represented by Amoxicillin + Clavulanate (2 g/day for 5 days) and Ibuprofen (800 mg/day for 3 days); rarely we used other variants in patients with allergies to these drugs. Short-term antibiotic therapy could not influence long-term dimensional parameters. In all patients, implants (various types) were applied to the area of the sinus lift and they received fixed dentures 6 months after the application of the implants. In only 4 sinuses, an amount of biomaterial was grafted at the time of applying the implants, considering the initial amount insufficient, as estimated from the ridge preparation.

### 2.3. Grafting Materials Used

We used a wide variety of biomaterials. All of them were homologated variants existing on the market. In almost all cases, we have used combinations of them. Also, in many cases, a small amount of autologous bone taken from the maxillary tuberosity was used. The biomaterials used are listed in Table 1.

### 2.4. Radiografic Measurements and Evaluation

Measurements were made using callipers on multiple orthopantomograms taken at different stages of the treatment, namely: T0—initial radiograph; T1—radiograph approximately 6 months after the sinus bone graft, before implant insertion; T2—radiography after a period of 6 months from the insertion of the implants, the moment when the patient returned for prosthetics; T3—radiograph taken post treatment, usually after several years.

In certain situations where the native crestal bone had a sufficient height to stabilize the implants (approximately 4–7 mm), the insertion of the implants was performed together with the SL grafting. Since the radiographs were taken at different times, on different devices, and obviously with slightly different positioning of the patient, a common denominator had to be found for all the radiographs of a patient. This common denominator was an unchanged element present on all radiographs of a patient and located as close as possible to the sinus on which the measurements were desired. We used, for example, the length of a canine remaining closed, the height of the mandibular body in an area, etc. This common element was considered in each case as the reference unit (1 U). Thus, all the measurements made vertically at the sinus level were percentages related to this unchangeable unit. The measurement error in millimeters was thus eliminated. 

**Figure 1:**
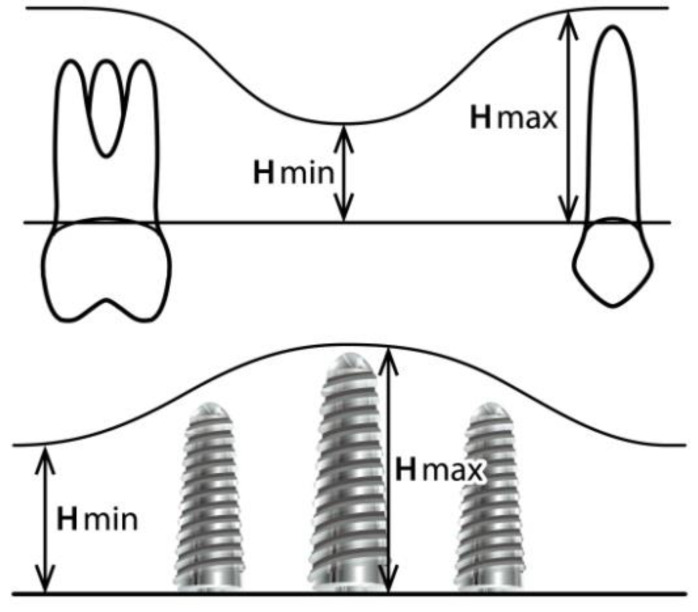
Initial and final measurements.

The minimum (H min) and maximum height (H max) of the area of interest (on which the implants were inserted) were measured vertically before intervention (T0), just before placing the implants (T1 or T2/upon the case) and also many years after the procedure (T3) (Figure 1).

Except for the measurements, the bone densities were determined on the initial native bone as well as on the grafted bone on the last radiograph.

To evaluate the bone density, we used a set of criteria developed and published in a study in 2008 [17]. That study was OPG compared to MDCT and clearly showed that radiological bone density can be assessed on panoramic radiographs with minimal errors according to clear criteria and with little observer self-calibration. Furthermore, in the maxilla, where the cortical bone is much less dense than in the mandible, the aspect of the trabeculae of the Haversian bone can be seen quite well. The density assessment criteria could be summarized as follows: density D1: compact, cortical bone; D2 density: dense bone with small intertrabecular spaces; D3 density: medium density bone, with wide intertrabecular spaces; D4 density: more radiolucent bone with very wide intertrabecular spaces; D5 density: radiolucent bone (no visible trabeculae).

The final aspect of the sinus new bone in its upper area was also evaluated and described.

The application of the biomaterial in the sinus is performed through the window in the side wall. It is impossible to control its shape if granules are used, as in our study. We would like to answer several questions: what is the shape of the upper limit of the biotransformed material over time? Does it remain as it was originally applied? Is there an evolution pattern? That is why we looked for various radiographs and found various aspects of the shape of the upper limit.

**Figure 2:**
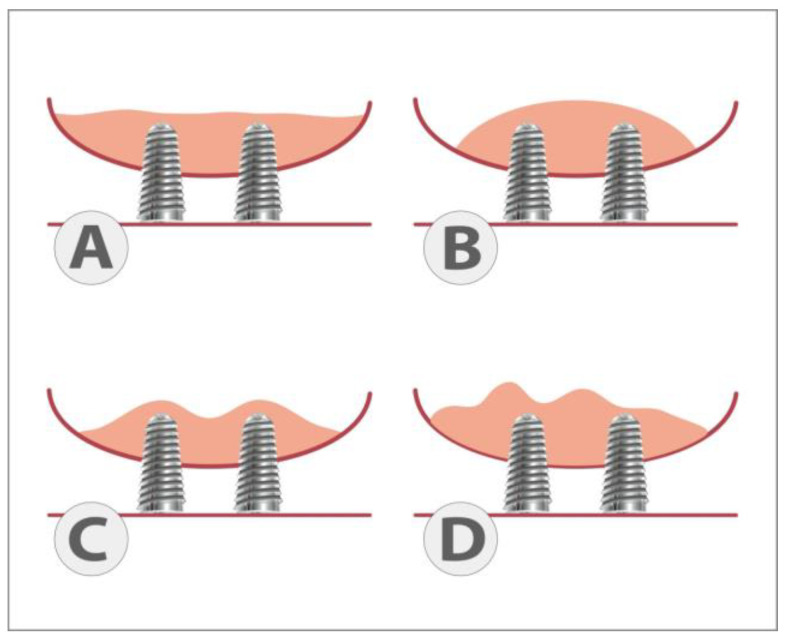
Different shapes of the upper limit of the biotransformed material over time. (**A**) Linear; (**B**) large dome; (**C**) micro-domes; (**D**) irregular.

Further, that is why we tried to approximate the shape of the upper limit with the following: (1) A large dome above all implants, a curved line with the concavity located below; (2) an approximately linear aspect from mesial to distal; (3) an irregular appearance, i.e., a line with multiple curves; (4) micro domes above each individual implant, a general linear appearance but with small elevations next to each implant (Figure 2).

In order to eliminate errors, the measurements, the assessment of the density, and the upper limit of the bone were made by several observers independently.

### 2.5. Statistics

For statistical analysis, we used the Microsoft Excel (version 2308, Redmond, WA, USA) and IBM SPSS Statistics 21 (Armonk, NY, USA). We used more tests. A *p* value below 0.05 was considered statistically significant.

Wilcoxon signed-rank test was used for the interpretation of radiological density data but also for the analysis of contractions. The Friedman test was used to follow the evolution over time of the H max and H min values, from T0 to T3. The Mann–Whitney test was used to compare the differences in annual graft contraction between genders. A Kruskal–Wallis H test was run to determine if there were differences in age between the groups according to the appearance of the top plane of the material. Sperman and Pearson associations were made between various parameters.

## 3. Results

In accordance with the inclusion criteria, 35 patients (20 men and 15 women), aged between 32 and 80 years, (with the average and the median age being 53) were enrolled in the group. There were 44 lateral sinus lift interventions. All patients were followed for a variable period of time between 0.5 and 11.6 years (with an average 4.7 years), between 2008 and 2022. All implants survived this period.

Many biomaterials were used. Each biomaterial was present in at least one sinus (Table 2).

**Figure 3:**
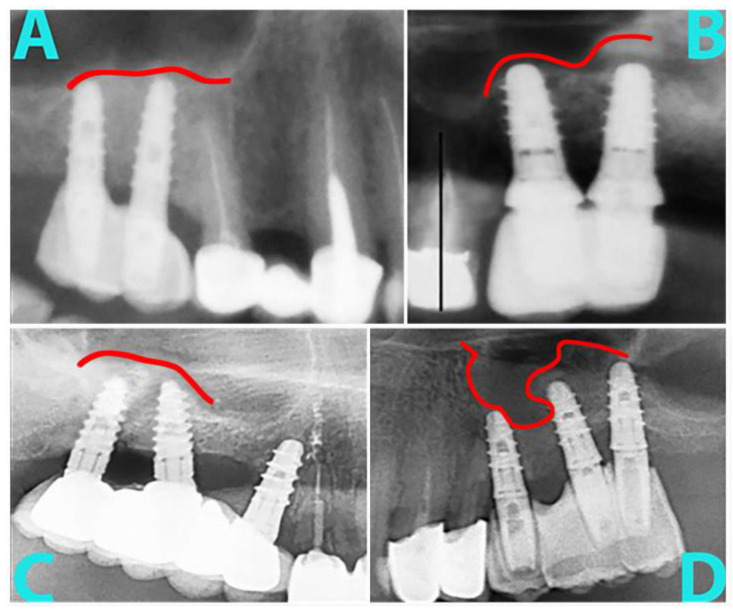
Various aspects of the upper bone plane: (**A**) relatively linear; (**B**) micro domes; (**C**) large dome; (**D**) irregular.

The first thing we evaluated was the shape of the upper limit of the biomaterial. Over time, it takes on the appearance of a large dome in most situations (36%), followed by a linear aspect in 32%, micro domes above the implants in 7%, irregular (23%), or impossible to appreciate on radiographs (2%). The main null hypothesis regarding the shape of the upper limit was rejected. See Figure 3 for some examples.

**Figure 4:**
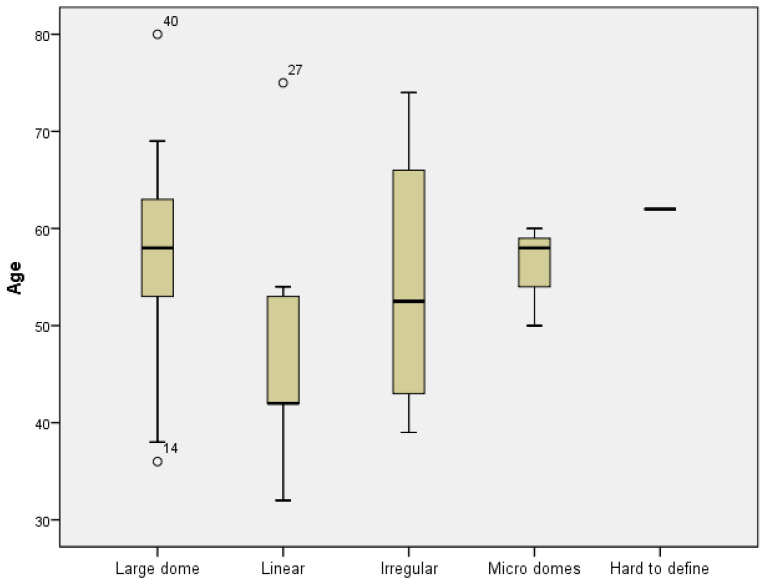
The appearance of the upper top of the graft in relation with the age of patient.

A Kruskal–Wallis H test was run to determine if there were differences in age between the five groups according to the appearance of the top plane of the material: large dome, linear, irregular, micro domes, and a hard-to-define shape. Distributions of ages were not similar for all groups, as assessed by visual inspection of a boxplot. The mean rank of age was not statistically significantly different between groups, χ^2^(4) = 8.034, *p* = 0.090. The null hypothesis was accepted as no significant differences were found between the groups regarding age (Figure 4).

Distributions of follow-up times were not similar for all mentioned groups, as assessed by visual inspection of a boxplot. The mean rank of the following up time was not statistically significantly different between groups, χ^2^(4) = 2.395, *p* = 0.664. The null hypothesis was accepted as no significant differences were found between the groups regarding following up time.

**Figure 5:**
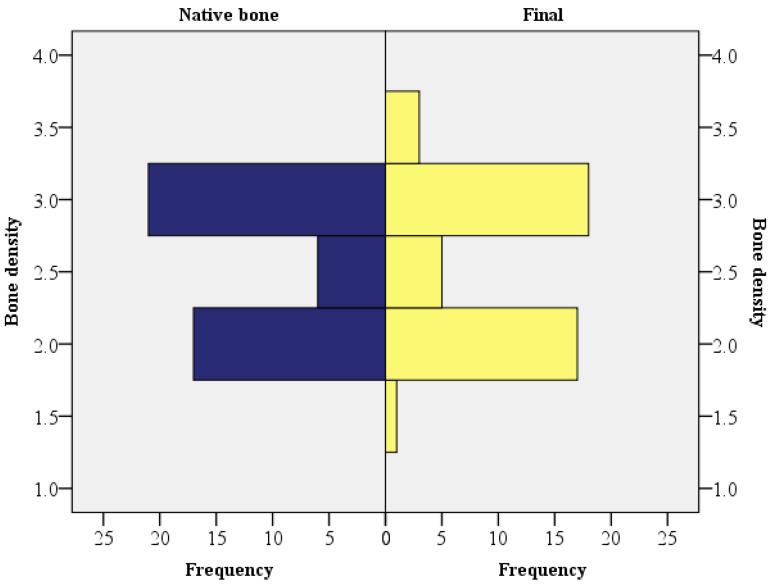
The native bone density and the final density of the grafted bone.

The second thing we evaluated was the radiological density. In most cases, the final radiological density of the grafted bone at T3 was similar to the one of the native bone (Z = −0.184, *p* = 0.854, using the Wilcoxon signed-rank). The second null hypothesis was retained (Figure 5).

**Figure 6:**
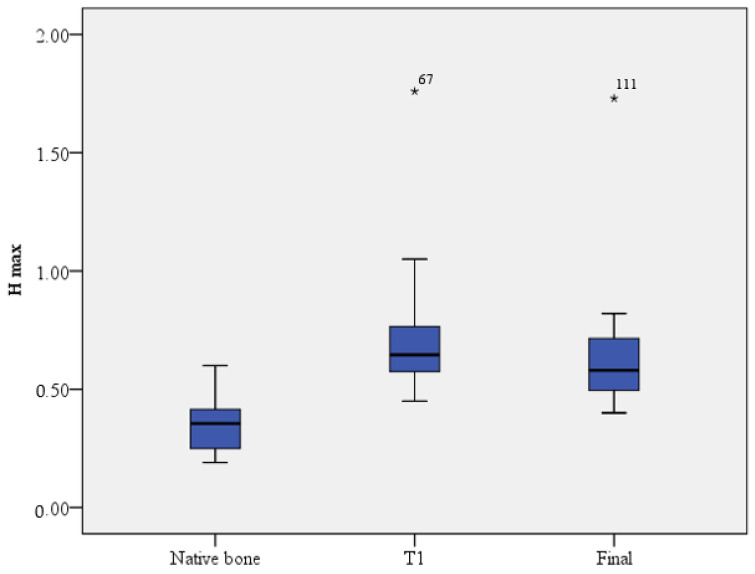
The evolution of H max at different time points.

The general efficiency of the sinus lift intervention can be appreciated by the final dimensional jump compared to the maximum height of the native bone. A Friedman test was run to determine if there were differences in H max measured at different times. Pairwise comparisons were performed with a Bonferroni correction for multiple comparisons. H max was significantly different at different time points, χ^2^(2) = 74.646, *p* ≤ 0.001. Post hoc analysis revealed statistically significant differences in H max from native bone (median = 0.35) to T1 (median = 0.64) (*p* < 0.001), from native bone to final H max (median = 0.58) (*p* < 0.001), and from T1 to final H max (*p* = 0.014). The same tendency was observed regarding H min (χ^2^(2) = 76.296, *p* ≤ 0.001). The third main null hypothesis was rejected (Figure 6).

**Figure 7:**
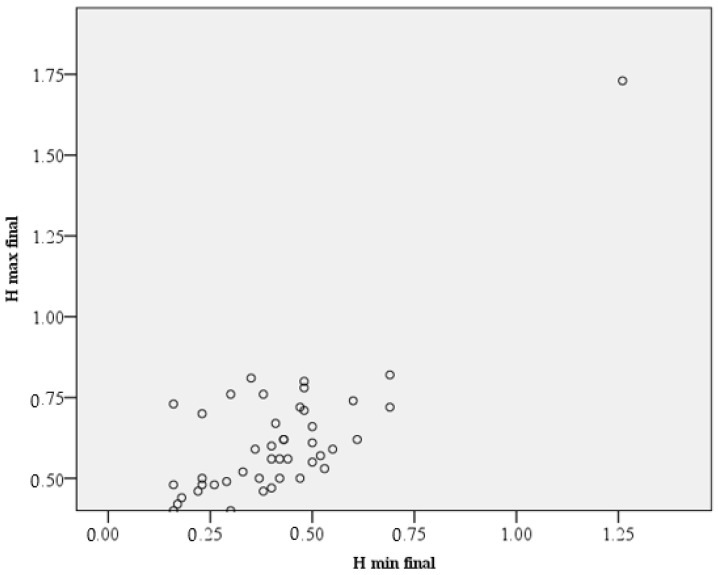
Correlation between H max and H min at T3. The values are pointed as small circles.

There is a statistically significant relationship between the final maximum height (H max final) and the final minimum (H min final) (rs = 0.545, *p* ≤ 0.01). For the native (initial) bone, the maximum and minimum values of its height were not statistically correlated. The bone may have any shape (*p* = 0.836). See Figure 7 for details.

**Figure 8:**
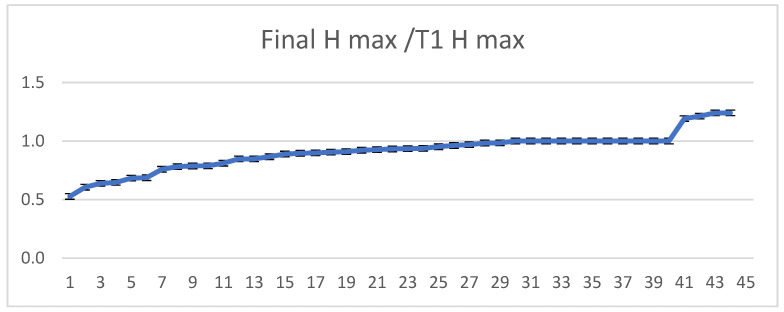
The ratio between the maximum height of the “stabilized” bone over time compared to the value from the moment of implant application, in ascending order. The x-axis shows the sinus current number; the y-axis shows the Final H max/T1 H max ratio.

The biomaterial undergoes a contraction phenomenon. Ideally, its size in years to come would be 1:1 compared to the size in T1. However, in clinical practice, in most cases, it was obtained in ratios below 1. As an exception, there were four cases in which the final H max was greater than H max T1 (>1). In the four cases, additional bone material was grafted at the time of implants insertion (Figure 8).

**Figure 9:**
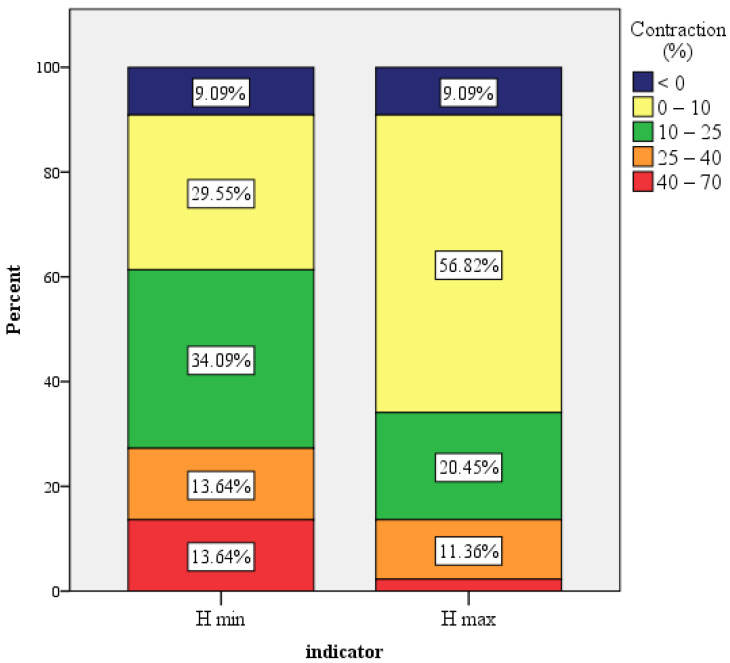
H min and H max contraction over time. The x-axis (indicator) shows the H min and H max contraction in columns; the y-axis shows the percentage of all cases that have a specific contraction (the contraction intervals have different colors).

The general H max contraction ranged from −24% and +47%, with a mean value of 8.6% and a standard deviation 0.15, CI: [0.03; 013]. The median value was 6.5%. In only one case, the contraction exceeded 40%. For a quarter of all the sinuses (*n* = 11), there was no contraction. The general H min contraction ranged from −55% and +70%, with a mean value of 17.3% (standard deviation 0.23, CI: [0.1; 0.24]). The median value was 14.4%. For only *n* = 7 cases, the contraction was equal to zero (Figure 9).

**Figure 10:**
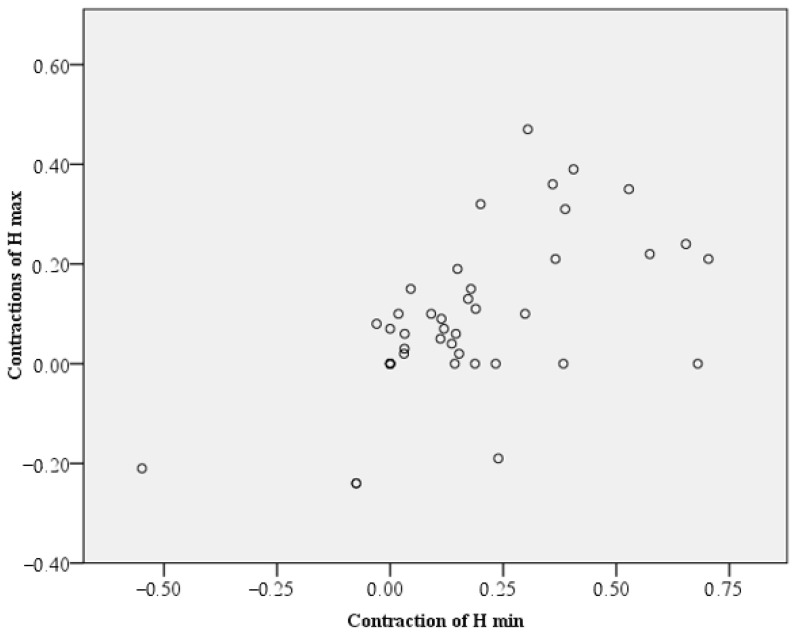
Correlation of H max and H min contractions, showing a positive value (+0.59 Spearman).

H min values suffered a significantly greater value contraction compared to H max values (Z = −2.937, *p* = 0.003, Wilcoxon test). This can be interpreted spatially through a tendency to curve the upper plane of the newly formed bone in time. For practical simplicity, we could notice that final H max was approximately 90% of the H max T1 and final H min was approximately 80% of the H min T1. However, there is a significant correlation (0.59 Spearman) between the contraction of H max and H min (Figure 10). This correlation does not have the maximum value of 1, as would be expected within the same material. Some factors could be involved, such as the shape and amount of native bone, which differ from one sinus to another.

The annual H max contraction ranged from −0.09 to 0.18 (−9% and 18%), with a mean value of 0.0267 (2.67%) (standard deviation 0.04, CI: [0.011, 0.041]). The median value was 0.018.

**Figure 11:**
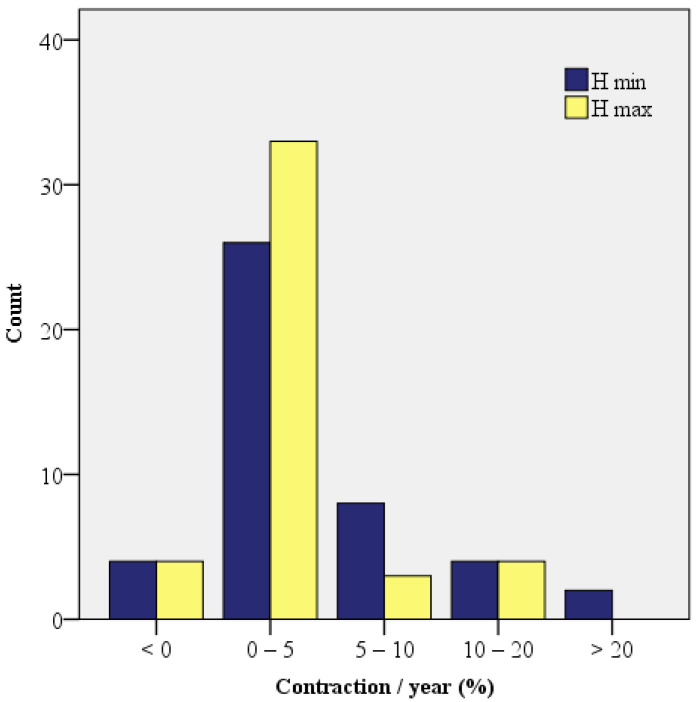
Histogram of the annual contractions.

The annual H min contraction ranged from −0.24 to +0.24 (−23.8% and +24.2%), with a mean value of 0.0433 (4.33%) (standard deviation 0.07, CI: [0.021, 0.065]). The median value was 0.0359 (half of the cases had a H min contraction of at most 3.59% per year. The H min/year contraction was significantly higher compared to the H max/year contraction (Z = −2.618, *p* = 0.009) (Figure 11).

There are no statistically significant differences between gender regarding annual contractions (Mann–Whitney U, *p* = 0.483, *p* = 0.642).

No significant correlation was found between age and annual contraction (Pearson correlation = −0.135 and Rho Spearman = −0.160).

## 4. Discussion

Our retrospective study was based on successful clinical cases in which a sinus bone graft (a wide variety of biomaterials) and inserted implants were followed over a long period of time. We wanted to study the common characteristics of these biomaterials regarding the aspect that the graft takes over time.

The image of the upper limit of the biomaterial was assessed two-dimensionally in the panoramic radiograph, as a mesio-distal aspect. Although the CBCT produces a more accurate three-dimensional image, its use is not justified without the suspicion of a pathology due to the higher radiation dose [18,19]. Panoramic radiography is necessary anyway before the insertion of the implants, after the osseointegration period and several years after the end of the treatment at a routine check-up.

Our proposed classification of the shape of the upper limit of the bone graft is a simple one, recognizable even by patients. Our study showed that the bone graft takes different forms over time (large dome, linear, micro domes, irregular, and hard-to-define) with a certain tendency to bend in the mesio-distal direction (due to differential shrinkage). There was no statistically significant correlation between the age of the subjects and a certain shape described, which suggests that the shape does not depend on age.

Appreciating the opacity of a thickness of X-rayed tissue can provide good information about what is on average there. This aspect has been neglected lately, but computed tomography can only provide strictly local information (in a point) regarding the radiological density, not summed, global information. Studies on radiological density are insufficient. One of them, regarding biomaterial of bovine origin, found that over time, the grey tones changed but the changes were declared “statistically insignificant” (for the first 7 months) [20]. We followed the cases for a longer period and we used a radiological bone density evaluation scale and highlighted the tendency of uniformity of the density of the graft according to the density of the native bone.

Bone biotransformation and remodelling also result in dimensional changes (so-called shrinkage). 

In our study, we preferred to express the dimensions and contractions of the biomaterial as a percentage, and to eliminate possible errors due to the patient’s position during the radiography. The measured contraction of the minimum size of the biomaterial (final H min) was on average 10% higher than the contraction of the maximum size (final H max) for the study group. The correlations of the maximum and minimum size of biotransformed material over time are statistically significant. The difference suggests that there are local factors involved in this dimensional change. The shape of the native bone, the size of the sinus, or other factors could be suspected. A 2015 study found no correlation between the size of the sinus and the dimensional changes of the biomaterial [21]. Further studies are needed to clarify all these aspects.

A very recent retrospective study (February 2023) similar to ours in terms of duration (patient follow-up between 2010–2019), also conducted using radiographs, concluded that the longer the time between the sinus graft and the implants’ insertion, the greater the shrinkage of the biomaterial. The remodelling of the grafted material was found to continue three years after the procedure. The average bone gain was 9.5 ± 3.47 mm and the shrinkage of the material was 1.57 ± 2.85 mm, which means approximately 16.5% [22].

A meta-analysis from 2021 concluded that a smaller graft shrinkage was associated with a smaller graft volume and a higher amount of new bone formation. The authors therefore suggested to minimize the quantity of used alloplastic grafts and xenografts [23].

We found that the process of remodelling continues for many years, according to other data from the literature [15]. The annual contraction can vary, being on average 2.67% for H max and 4.33% for H min, without statistically significant differences between different ages or genders. It is to be expected that the decrease in bone mass is slow, but it continues throughout life. The fear that over time, the physiological decrease in bone mass becomes unable to support the implants, is not justified for the period discussed, but longer studies are recommended to have a clearer picture.

Some studies suggest that the major dimensional changes happen in the first 6 months [14]. This could explain why, in the four cases in which we supplemented the graft at the time of implants insertion, higher values of the maximum height were obtained over time.

It seems that, regardless of the height of the original native bone, it is important how much the biomaterial is grafted so that it remains sufficient over time.

The biggest limitation of this study is the number of cases that should be expanded for a more correct understanding and appreciation of the phenomena. Also, the use of the panoramic radiography instead of CBCT offers limited possibilities for dimensional measurements. We believe that this study, with all its limitations, could still be in daily practice an analytical witness regarding the evolution of the biomaterials that we used for sinus grafting.

## 5. Conclusions

Our study showed that the shape of the top plane of the grafted and bio-transformed material can take the form of a large dome, a mesio-distal line, an irregular shape or the shape of microdomes above the implants in most cases. The applied biomaterial contracted over time, mostly at the level of its minimum height, which suggests the tendency of the upper edge to curve. The additional application of biomaterial together with the implants seems to have a beneficial effect on the final vertical dimension of the bio-transformed material. The final dimensions of the graft seem to be independent of the dimensions of the native bone but correlated with the amount of grafte biomaterial. Age and gender made no difference in graft dimensional changes over time.

## Figures and Tables

**Table 1:** The biomaterials used in the study.

Biomaterial	Form	Origin	Type
Bio-Oss (Geistlich, Wolhusen, Switzerland)	Granules 1–2 mm	Bovine Matrix	Xenograft
SintLife (Finceramica, Faenza RA, Italy)	Microgranules	hydroxyapatite and Mg^2+^	alloplastic
Cerasorb (Curasan, NC, USA)	granules 1000–2000 microns	Beta-TCP	alloplastic
Gen-Os (OsteoBiol, Torino, Italy)	granules 1000–2000 microns	porcine origin	allograft
Sterling (Regeneration Technologies Inc., Neunkirchen, Germany)	cancellous chips	bovine bone matrix	xenograft
Tutobone (RTI Surgical, Neunkirchen, Germany)	granulates 2–4 mm	bovine origin bone	xenograft
Smartbone (IBI, Mezzovico, Switzerland)	block and granulates 2–4 mm	bovine mineral bone matrix, bioresorbable polymer and collagen	xenograft + alloplastic
DBM (Dizg, Berlin, Germany)	granules 1–3 mm	demineralized bone matrix, human origin	allograft
Osteoxenon (BioTeck, Vicenza, Italy)	cancellous 0.5–1 mm	equine origin bone with preserved type 1 collagen	xenograft
Cerabone (Biotiss, Zossen, Germany)	granules 1–2 mm	bovine origin	xenograft
Bio-Gen (Bioteck, Vicenza, Italy)	granules 500–1000 µm	equine origin	xenograft

**Table 2:** The distribution of biomaterials in sinuses and the average follow-up for each biomaterial.

Biomaterial	The Current Number of the Sinus	Average Follow-Up
DBM (DIZG)	16, 17, 25, 26, 28, 29, 33, 34	4.3 years
Sterling	7, 11, 12, 13, 15, 19, 20, 23, 41, 42	5.1 years
Bio-Oss	1, 6, 10, 14, 30, 32, 39, 40	5.0 years
Bio-Gen	5	3.9 years
Tutobone	38	2.7 years
Smartbone	37	2.7 years
Gen-Os	5, 23	4.3 years
Osteoxenon	43, 44	9 years
Cerabone	43, 44	9 years
SintLife	2, 3, 5, 8, 21, 22, 24	4.6 years
Cerasorb	1, 4, 6, 9, 14, 17, 18, 25, 26, 27, 31, 32, 35, 36, 39	3.8 years

## Data Availability

The data presented in this study are available on request from the corresponding author.
